# Amino Acid Chelated Iron Versus Ferric Ammonium Citrate on Iron Status in Egyptian Children with Iron Deficiency Anemia: A Randomized Controlled Study

**DOI:** 10.1007/s12288-024-01746-6

**Published:** 2024-04-24

**Authors:** Hanan Hamed, Ola M. Abdel Samie, Ayat A Motawie, Manal E Kandil, Gamila S. M. El-saeed, Nehal Abdelhamid

**Affiliations:** 1 Pediatrics Department, National Research Centre, Dokki, 12622 Giza, Cairo Egypt; 2 Medical Biochemistry Department, National Research Centre, Dokki, Giza, Cairo Egypt; 3 Child Health Department, National Research Centre, Dokki, Giza, Cairo Egypt

**Keywords:** Iron amino acid chelated, Ferric ammonium citrate, Iron deficiency anemia

## Abstract

Few studies have compared the relative effectiveness of different iron compounds on iron status in school-age children with iron deficiency anemia. The objective of this study was to compare the effect of iron amino acid chelated (AACI) preparation versus ferric ammonium citrate (FAC) in treatment of iron deficiency anemia. Patients and methods: A randomized, clinical study was conducted on one hundred and sixty children aged 5–13 years old proved to have iron deficiency according to guide lines of WHO, 2001. All included children were subjected to the following laboratory investigations: CBC, reticulocytic count, CRP, serum iron, total iron binding capacity (TIBC), serum ferritin (SF), and serum hepcidin. Patients were assigned to two treatment regimens on randomized base 1:1 either to supplement with (AACI) or (FAC) once daily at bedtime. The subjects were followed up for eight weeks. Results: At the end of the study, group 1 who received AACI had increase in: Hb from 9.9 ± 1.1to 11.5 ± 0.3 gm/l(*p* = 0.01), MCV(fl) from 63.57.7 ± to 69 ± 6.3 (*p* = 0.05), serum iron from 49.5 ± 5.8 to 87 ± 12.7ug/dl(*p* = 0.001), serum ferritin from26.2 ± 10.5to 116.4 ± 19.7ng/ml(*p* = 0.001) while in group 2 who received FAC, there was increase in: HB from10.1 ± 1.7 to 11.2 ± 0.8, (*p* = 0.1), MCV from 64.5 ± 8.02 to 73.2 ± 8.9 (*p* = 0.01), serum iron from 48.2 ± 3.5to74 .3 ± 15 ug/dl (*p* = 0.01) and serum ferritin from 28.1 ± 9.3 to 84.3 ± 15.2 ng/ml (*p* = 0.006). Conclusion: Our study showed much improvement in iron status indices in AACI preparation with no significant statistically difference between it and FAC.

## Introduction

There are many different oral iron supplementations like ferric ammonium citrate (FAC), ferrous sulfate, iron amino acid chelated preparations, ferrous bis-glycinate, ferric glycine and others. All tend to have different pharmacokinetics and pharmacodynamics which alter their medical influence [1–3].

Studies found preferable performance of iron amino acid chelated preparations over ferrous salts concerning the serum ferritin levels [4, 5].Their explanation was that, iron bis-glycinate chelate does not form insoluble compounds with tannins, oxalactes, and phytates (highly present in the cereal diets of the poor developing countries), therefore it has a much better bioavailability [6]. Szarfarc et al., reported that the amino acid chelated iron was much better than ferrous salts in the treatment of IDA, due to higher bioavailability (4–7 times more) and that its efficacy was three times more than ferrous salts even at low dose and with a short course of time [7].

In some interesting researches, it was found that although the absorption of ferric ammonium citrate (FAC) was significantly lower, still it had an appreciable and adequate iron bioavailability. In fact, the relative bioavailability (RBV) of iron in the ferric iron salts vary with the composition of the meals and the age of the consumer [8, 9].

Here it is very much worth reporting that some studies declared that, the iron from ferric ammonium citrate is much better absorbed when consumed at relatively low pharmacological doses and that citric acid can increase iron absorption, up to two to three folds when present at quantities as high as 1 gm. Accordingly, the citrate in the ferric ammonium cirate might have made some enhancement in the absorbability of the ferric iron with consequent improvements in the haematological parameter [10]. Ferrous salt is the most commonly used iron compound in supplementation programs because of its efficiency and low cost [11].

So, in this study, we aimed to compare between an iron amino acid chelated preparation and a conventional iron salt ferric ammonium citrate (FAC) as regards the efficacy in correcting IDA in children.

## Patients and Methods

**Fig. 1:**
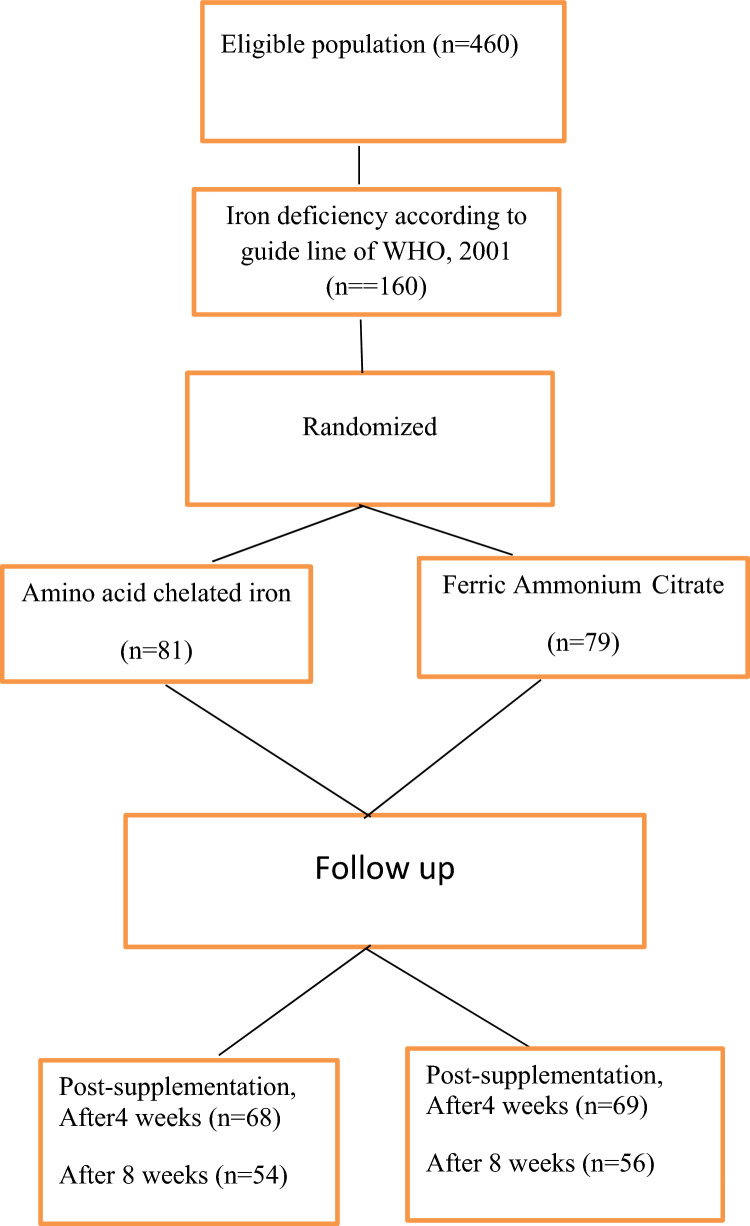
Study population. Flow of patients through the study

A randomized, clinical study was conducted on one hundred and sixty children aged 5–13 years old (Fig. 1).The children proved by laboratory investigations to have iron deficiency according to WHO guide lines, 2001 [12] (serum ferritin ≤ 30 mg/l, transferrin saturation ≤ 16%, MCV ≤ 75 fl and MCHC ≤ 32 g/l) an anemic with hemoglobin level ≤ 11 g/dL adopted by Egypt Demographic and Health Survey (EDHS, 2015) [13].

The children were recruited from pediatrics clinics, National Research Centre (MREC) in the period from January 2019 to December, 2019. The children were from the same socioeconomic classes with similar diet habits resumed from history.

Exclusion criteria were: chronic infections, chronic inflammatory diseases (rheumatic disease, inflammatory bowel disease), parasitic infestation, thalassemia traits A and B, iron supplementation and blood transfusion during the last 6 months. All the included children were subjected to full history taking (with stress on dietetic history, iron supplementation during last 6 months and manifestation of iron deficiency (fatigue and diminished capability to perform hard work, leg cramps on climbing stairs, poor school performance, and reduced resistance to infection) and full clinical examination with stress on (pallor of the mucous membranes, koilonychia, a glossy tongue and angular stomatitis). Venous blood samples were withdrawn from 10 to 11am (to avoid diurnal variation), 1 ml was collected into EDTA vacutainer tubes (for CBC and reticulocytic count) and 3 ml were collected into plain tubes and centrifuged. Serum samples were stored at -80^ °C^ for further laboratory investigations.

## Laboratory Investigations

Complete blood count (using automated hematology analyzer Sysmx XN100 **(**Sysmex America Inc), reticulocytic count (using automated hematology analyzer Sysmx XN100i (Sysmex America Inc), CRP (using IMMUNOSPEC REFE 29–056) (Bioquote Ltd, UK), serum iron and total iron binding capacity (TIBC) (using Olympus AU400(Autoanalyzer, Japan).Serum ferritin (SF) (Biocheck, Inc;Cat. No. EC-1025) BioCheck, Inc.

Serum hepcidin by ELISA (Cat No: E1019 Hu) (Bioassay Technology Lab, China).

Transferrin saturation % was calculated using the following formula (TS% = serum iron /TIBC × 100 (WHO, 2001) [11].

Children with iron deficiency anemia were assigned to two treatment regimens on randomized base 1:1 to either supplements with Amino acid chelated iron, (10 mg elemental iron /sachet) or Ferric Ammonium Citrate, (10 mg elemental iron /5 ml) once daily at bedtime. The dose of either therapy was 10 mg elemental iron /day for 8 weeks according to Moretti et al., 2015[14] and Schrier, 2015 [15] who used this dose as the standard dose.

Full history taking, including adverse effects (abdominal colic, diarrhea, vomiting and constipation), complete physical examination and all laboratory investigations were repeated twice (4 and 8 weeks) from iron therapy.

## Ethical Approval

Before the study, the guardians of all patients were informed about the contents & type of the study and informed written consents were obtained from all of them. The study was approved by the medical research ethical committee of National Research Center (reference No. 16304).according to the Institutional Committee for the Protection of Human Subjects and adopted by the 18th World Medical Assembly, Helsinki, Finland.

## Statistical Analysis

Statistical package for the social sciences (SPSS, version 15 for Windows; SPSS Inc., Chicago Illinois, USA) was used for statistical analyzed.Continuous variables were expressed as means ± Standard deviation (SD). Comparison of numerical variables between the study groups was done using independent t-test. Paired sample t-test was used to compare means of 2 variables (pre and post-iron therapy) within a group. The p-values ≤ 0.05 were taken as significant.

## Results

The anemic patients were classified according to the type of iron supplementation into two groups: group 1 who received Amino acid chelated iron and group 2 who received Ferric Ammonium Citrate. Table 1 showed that the pretreatment values of hemoglobin, red cell indices, serum iron, TIBC, TS%, serum ferritin and serum hepcidin were comparable for both groups. After 4 and 8 weeks of iron treatment, no significant difference was found between both groups as regards hemoglobin, red cell indices, serum iron, TIBC, TS%, serum ferritin and serum hepcidin.

**Table 1:** Comparison of the hematologic parameters at baseline, after 4 and 8 weeks of treatment in the two studied groups

Parameters		Group 1 (IAAC)	Group 2 (FAC)	P value
Number		81	79	
Age (years)		10.9 ± 3.3	10.2 ± 3.2	N.S
Sex (male/female)		38/43	36/43	N.S
BMI (Kg/m^2^)		17.4 ± 4.5	18.5 ± 4.2	N.S
RBCS	Baseline	4.5 ± 0.41	4.6 ± 0.6	0.2
	After 4 weeks	4.8 ± 0.6	4.7 ± 0.8	0.1
	After 8 weeks	5.2 ± 0.41	5.1 ± 1.3	0.7
HB(gm/L)	Baseline	9.9 ± 1.1	10.1 ± 1.7	0.4
	After 4 weeks	10.43 ± 0.5	10.41 ± 1.1	0.5
	After 8 weeks	11.5 ± 0.3	11.2 ± 0.8	0.6
HCT%	Baseline	31.45 ± 3.4	32.4 ± 3.9	0.9
	After 4 weeks	32.9 ± 8.4	32.7 ± 4.9	0.2
	After 8 weeks	34.07 ± 2.8	34.8 ± 3.3	0.5
MCV(fl)	Baseline	63.5 ± 7.7	64.5 ± 8.02	0.5
	After 4 weeks	68.2 ± 6.9	67. ± 7.7	0.8
	After 8 weeks	69.6 ± 6.3	73.2 ± 8.9	0.06
MCH(pg)	Baseline	19.91 ± 3.5	20.9 ± 3.1	0.06
	After 4 weeks	21.2 ± 3.8	24.1 ± 3.9	0.4
	After 8 weeks	22.9 ± 3.1	24.9 ± 3.7	0.06
MCHC(g/dl)	Baseline	30.2 ± 4.7	30.5 ± 6.1	0.3
	After 4 weeks	30.6 ± 1.3	31.5 ± 2.8	0.8
	After 8 weeks	30.9 ± 8.3	33.7 ± 2.4	0.08
RDW%	Baseline	14.8 ± 2.3	15.8 ± 3.4	0.6
	After 4 weeks	14.63 ± 4.9	14.70 ± 2.6	0.6
	After 8 weeks	14.5 ± 2.4	12.3 ± 1.6	0.01
Serum iron(ug/dl)	Baseline	49.5 ± 5.8	48.2 ± 3.5	0.7
	After 4 weeks	69.2 ± 18.8	53.2 ± 14.04	0.4
	After 8 weeks	87 ± 13.7	74.3 ± 15	0.4
Serum TIBC(ug/dl)	Baseline	385.5 ± 45.5	400.2 ± 68.1	0.1
	After 4 weeks	290.50 ± 40.6	349.00 ± 78.2	0.08
	After 8 weeks	264.04 ± 69.4	297.7 ± 28.1	0.1
Transferrine saturation%	Baseline	14.7 ± 1.8	14.8 ± 2.7	0.2
	After 4 weeks	29.8 ± 7.9	17.3 ± 2.7	0.1
	After 8 weeks	38.9 ± 6.7	25.6 ± 5.2	0.05
Serum ferritin(ng/ml)	Baseline	26.2 ± 10.5	28.1 ± 9.3	0.4
	After 4 weeks	68.8 ± 19.5	42.06 ± 14.4	0.5
	After 8 weeks	116.4 ± 19.7	84.3 ± 15.2	0.3
Serum hepcidin(pg/ml)	Baseline	293.5 ± 60.3	307.3 ± 60.4	0.3
	After 4 weeks	433.35 ± 87.8	360.73 ± 77.3	0.7
	After 8 weeks	407.4 ± 84.5	366.5 ± 57.5	0.5

Group 1 (who receive Amino acid chelated iron). Group 2 (who receive Ferric Ammonium Citrate)

RDW showed no statically difference at base line and after 4 weeks of treatment while after 8weeks it showed significant statistical difference (*p* = 0.01) with much decrement in group 2.

Table 2 showed that group 1 (who received Amino acid chelated iron), had increase in Hb from 9.9 ± 1.1 to 11.5 ± 0.3 gm/l (*p* = 0.01), increase in serum iron from 49.5 ± 5.8 to 87 ± 12.7ug/dl(*p* = 0.001), increase in serum ferritin from 26.2 ± 10.5to 116.4 ± 19.7ng/ml(*p* = 0.001) while in group 2 ( who received Ferric Ammonium Citrate), the HB increased from 10.1 ± 1.7 to 11.2 ± 0.8, (*p* = 0.1), serum iron increased from 48.2 ± 3.5to74.3 ± 15 ug/dl (*p* = 0.01) and serum ferritin from 28.1 ± 9.3 to 84.3 ± 15.2 ng/ml (*p* = 0.006) (Table 2).

**Table 2:** Hematologic parameters at base line and after 8 weeks of treatment in each group separately

Parameters		Group 1 (IAAC)	Group 2 (FAC)
RBCS	Baseline	4.5 ± 0.41	4.6 ± 0.6
	After 8 weeks	5.2 ± 0.41	5.1 ± 1.3
	P value	0.007	0.04
HB(gm/L)	Baseline	9.9 ± 1.1	10.1 ± 1.7
	After 8 weeks	11.5 ± 0.3	11.2 ± 0.8
	P value	0.01	0.1
HCT%	Baseline	31.45 ± 3.4	32.4 ± 3.9
	After 8 weeks	34.07 ± 2.8	34.8 ± 3.3
	P value	0.003	0.02
MCV(fl)	Baseline	63.5 ± 7.7	64.5 ± 8.02
	After 8 weeks	69.6 ± 6.3	73.2 ± 8.9
	P value	0.05	0.01
MCH(pg)	Baseline	19.91 ± 3.5	20.9 ± 3.1
	After 8 weeks	22.9 ± 3.1	24.9 ± 3.7
	P value	0.2	0.5
MCHC(g/dl)	Baseline	30.2 ± 4.7	30.5 ± 6.1
	After 8 weeks	30.9 ± 8.3	33.7 ± 2.4
	P value	0.5	0.1
RDW%	Baseline	14.8 ± 2.3	15.8 ± 3.4
	After 8 weeks	14.5 ± 2.4	12.3 ± 1.6
	P value	0.9	0.002
Serum iron(ug/dl)	Baseline	49.5 ± 5.8	48.2 ± 3.5
	After 8 weeks	87 ± 13.7	74.3 ± 315
		0.001	0.01
Serum TIBC(ug/dl)	Baseline	385.5 ± 45.5	400.2 ± 68.1
	After 8 weeks	264.04 ± 69.4	297.7 ± 28.1
	P value	0.6	0.6
Transferrin saturation%	Baseline	14.7 ± 1.8	14.8 ± 2.7
	After 8 weeks	38.9 ± 6.7	25.6 ± 5.2
	P value	0.001	0.001
Serum ferritin(ng/ml)	Baseline	26.2 ± 10.5	28.1 ± 9.3
	After 8 weeks	116.4 ± 19.7	84.3 ± 15. 2
	P value	0.001	0.006
Serum hepcidin (pg/ml)	Baseline	293.5 ± 60.3	307.3 ± 60.4
	After 8 weeks	407.4 ± 84.5	366.5 ± 57.5
	P value	0.03	0.64

Group 1 who receive iron Amino Acid Chelated (IAAC). Group 2 who receive Ferric Ammonium Citrate (FAC)

Collectively Tables 1, 2 showed that each group separately improved in iron indices and hematological parameters with no significant difference between the two groups.

Serum hepcidin increased significantly after 4 weeks of iron therapy in both groups but after8 weeks of treatment it decreased but did not reach the base line.

The adverse effects in form of colicky abdominal pain, diarrhea or constipation were higher in group 2 however it was not significant between the two groups.

## Discussion

In the current study, a comparison was held between FAC and IAAC as regards their efficacy, tolerability and cost, in treatment of IDA in children. Our study showed significant increase in HB level in children with Iron amino acid chelated (IAAC), however there was no difference between the two groups that agreed with Santos et al., and Bagna et al., [11, 16].

Red cell distribution width (RDW) started to decrease in both groups of anemic children after the initiation of iron treatment. This decrease, that indicates improvement, was quite obvious in both groups after 4 weeks of treatment but without any significant statistical difference between the 2 groups. However, after 8 weeks of that same treatment, the decrements, varied where group 2 anemic children showed much more declination in the RDW than group 1 and there was a statistically significant difference (*P* = 0.01). This difference may be due to occurrence of reticulocytosis after iron treatment in some cases [17]. Bayukkaragoz et al., reported that RDW may need longer time to get to the normal level though other haematological indices became similar to control [18].

There was a significant improvement in serum iron in both groups without significant difference between the two groups, however the improvement is much more in group 1.In accordance with our study, some researchers declared that amino acid chelated iron had much better absorption, bioavailability and safety profile [15, 16].

Transferrin saturation percentage was significantly higher in group I than in group 2 after 8 weeks of treatment. This ensures that the amino acid chelated iron was far better than FAC in improving the serum iron and accordingly there was an increase in the transferrin iron-binding sites that were occupied [15, 16].

Our results showed significant improvement in serum ferritin without significant difference between the two groups, however the improvement in group 1 was better. Chemical composition of IAAC (ferrous cation coupled with two glycine molecules) makes it partially resistant to enteric enzyme action, binding action of metals, phenols and food dietary fibres with which iron can form insoluble compounds. Likewise, the amino acids bonding allows less direct exposure of iron to the GIT mucosal cells and this in turn decreases the side effects and the local toxicity [6]. On other hand, the formation of unabsorbable insoluble ferric hydroxides in the duodenum is the reason why the absorption of FAC is usually significantly lower [10].

Regarding the hepcidin levels in our study, we found out that its level shooted up after 4 weeks of iron supplementation treatment. This was rational because hepcidin plays a pivotal role in iron homeostatsis [19]. Similarly, other studies reported that hepicidin levels get acutely elevated with oral iron supplements. Investigations are going on concerning the duration and extent of that uprise, its effect on iron absorption and its dependence on the dose of the administered iron [20, 21].

After 8 weeks of treatment, the hepcidin levels decreased where the conditions returned more or less to the normal state. In IDA, the drop in the levels of iron-bound transferrin triggesr hepcidin suppression. In turn, this suppression leads not only to uprise in iron absorption but also to release of the recycled iron from the splenic macropahges into the circulation [22].

It is very much worth reporting that low levels of hepcidin facilitates absorption of iron, and that is why low dose oral iron supplementation (10mg elemental iron/day) correct IDA in most patients [23].

## Conclusion

Our study showed that low dose of iron (10mg elemental iron/day) with either the two regimen of treatment improved hemoglobin level and iron status indices which is much more in amino acid chelated iron preparation with no significant statically difference between it and Ferric Ammonium Citrate. However, the price of iron amino acid chelated (AACI) preparation is double the price of ferric ammonium citrate.

Our results in concert with other literatures, they should help stimulate the conduct of further clinical trials evaluating lower and less frequent dosing of oral iron.

## Recommendation

A large study with longer period of time is needed to show the effect of low dose supplementation with Ferric Ammonium Citrate versus Amino acid chelated iron on iron status in children with iron deficiency anemia to verify this results.

## References

[1] Tiwari M, Dubey V, Srivastava IV (2021) Comparative study of Different Oral Iron Preparations in females of Reproductive Age Group. Journal of pharmaceutical Research international 33(28B):93–100

[2] Usha R (2000) Functional consequences of nutritional anemia during pregnancy and early childhood. In: Nutritional anemias. CRC Press, Taylor & Francis Group, pp 43–66

[3] Allen LH (2000) Anemia and Iron deficiency: Effects on pregnancy out come. Am J Clin Nutr 71(5 Suppl):1280S-S1284. 10.1093/ajcn/71.5.1280s 10799402 10.1093/ajcn/71.5.1280s

[4] Gupta N, Gupta R, Juhan U, Bais A (2020) Comparative study of aifferent oral iron preparations in Gynecological and postnatal patients. International Journal of clinical obstetrics and Gynecology 4(4):330–332

[5] Pineda O, Ashmead HD (2001) Effectiveness of treatment of iron deficiency anemia in infants and young children with ferrous bis- glycinate chelate. Nutrition 17(5):381–384 11377130 10.1016/s0899-9007(01)00519-6

[6] Ximena D, Martinez H, vilchis- GilJ, et al (2014) Effect of supplementation with ferrous sulfate or iron bis – glycinate chelate on ferritin concentration in Mexican schoolchildren: a randomized controlled trial. Nutr J 1(13):71 10.1186/1475-2891-13-71 PMC4107593 25023784

[7] Szarfarc De Marinal S, cassana N, et al (2001) Relative effectiveness of iron bis- glycinate chelate (ferrochel) and ferrous sulfate in the control of iron deficiency in pregnant women Archivos Latinoamericanos de Nutricion. ALAN V 51(1):1 11688081

[8] Chavasit V, Porasuphatanas SU et al (2015) Iron Bioavailability in 8–24 months- old that children from a micronutrient – fortified quick – cooking rice containing ferric ammonium citrate or a mixture of ferrous sulphate and ferric sodium ethylene diaminetetraacetic acid. Matern Child Nutr 11(4):179–187 25721887 10.1111/mcn.12167 PMC6860205

[9] Hurrell RF, Egli A (2010) Iron bioavailability and dietary reference values. Am J Clin Nutr 91:1461S-1467S 20200263 10.3945/ajcn.2010.28674F

[10] Walczykt TS, Zeder C et al (2005) Iron absorption by human subjects from different iron fortification compounds added to thai fish sauce. Eur J Clin Nutr 59:668–674 15756294 10.1038/sj.ejcn.1602125

[11] Santos M, Nogueira N, Silva DA (2007) Effectiveness of different iron supplementation strategies on hemoglobin and ferritin levels among school children in Teresina. Piaui State Brazil. Cad Saude Publica 23(7):1547–1552 17572803 10.1590/s0102-311x2007000700005

[12] WHO (2001) Iron deficiency: assessment, prevention, and control. A guide for programme managers, Geneva, World Health Organization

[13] El-Zanaty and Associates [Egypt], and ICF International (2015) Egypt Demographic and Health Survey 2014. Ministry of Health and Population and ICF International, Cairo, Egypt and Rockville, Maryland, USA

[14] Moretti D, Goede JS, Zeder C, Jiskra M, Chatzinakou V, Tjalsma H et al (2015) Oral iron supplements increase hepcidin and decrease iron absorption from daily or twice-daily doses in iron-depleted young women. Blood 126:1981–9 26289639 10.1182/blood-2015-05-642223

[15] Schrier SL (2015) So you know how to treat iron deficiency anemia. Blood 126:1971 26494915 10.1182/blood-2015-09-666511

[16] Bagna R, Spada E, Coscia A (2018) Efficacy of Supplementation wth iron sulphate corapared to Iron Bisglycinate chelate in pneterm infants. Curr Pediatr Rev 14(2):123–129 29366419 10.2174/1573396314666180124101059 PMC6416193

[17] Kriptani A, Sharma A, Radhika AG et al (2016) FOGSI general clinical practice recommendations of iron deficiency in adolescent girls. Management of iron deficiency in adolescent girls. 10.13140/RG.2.2.3/093-78564

[18] Bayukkaragoz B, Akgun NA, Bulus AD et al (2017) Can soluble transferrin receptor be used in diagnosing IDA and assessing iron response in infants with moderate acute malnutrition. Arcjovps Argemtomps de Pediatria 115(2):125–132 10.5546/aap.2017.eng.125 28318177

[19] Rojas ML, Sánchez J, Villada Ó, Montoya L, Díaz A, Vargas C et al (2013) Effectiveness of iron amino acid chelate versus ferrous sulfate as part of a food complemen in preschool children with iron deficiency. Biomedica 33:350–60 24652170 10.7705/biomedica.v33i3.775

[20] Choi HS, Song Sh, Lee JH et al (2012) Serum Hepcidin levels and iron parameters in children with iron deficiency. The Korean Journal of Hematology 47(4):286–292 23320008 10.5045/kjh.2012.47.4.286 PMC3538801

[21] Mahajan G, Sharma S, Chandra J, Nanjia A (2017) Hepcidin and iron parameters in children with anemia of chronic disease and iron deficiency anemia. Blood Res 52(3):212–217 29043237 10.5045/br.2017.52.3.212 PMC5641514

[22] Camaschella C (2015) Iron Deficiency: New Insights into diagnosis and treatement. Hematology 1:8–13 10.1182/asheducation-2015.1.8 26637694

[23] Capellini MD, Musallam KM, Taher AT (2019) Iron deficiency anemia revisited. J Intern Med 287(2):153–170 31665543 10.1111/joim.13004

